# Supplementary material for: ppGpp accumulation reduces the expression of the global nitrogen homeostasis-modulating NtcA regulon by affecting 2-oxoglutarate levels
Source: Commun Biol. 2023 Dec 25;6:1285. doi: 10.1038/s42003-023-05632-1 (PMC10749895; doi:10.1038/s42003-023-05632-1)
Supplement: Supplementary file 2 — Supplementary Information [file 42003_2023_5632_MOESM2_ESM.pdf]

Supplementary Information for

ppGpp accumulation reduces the expression of the global nitrogen homeostasis-modulating NtcA regulon by affecting 2-oxoglutarate levels

Ryota Hidese, Ryudo Ohbayashi, Yuichi Kato, Mami Matsuda, Kan Tanaka, Sousuke Imamura, Hiroki Ashida, Akihiko Kondo, and Tomohisa Hasunuma*.

*Corresponding author: Tomohisa Hasunuma

Email: hasunuma@port.kobe-u.ac.jp

This PDF file includes:

Supplementary Figure 1-6

Supplementary Table 1

Other supplementary materials for this manuscript include the following:

Supplementary Data 1
Supplementary Data 2
Supplementary Data 3

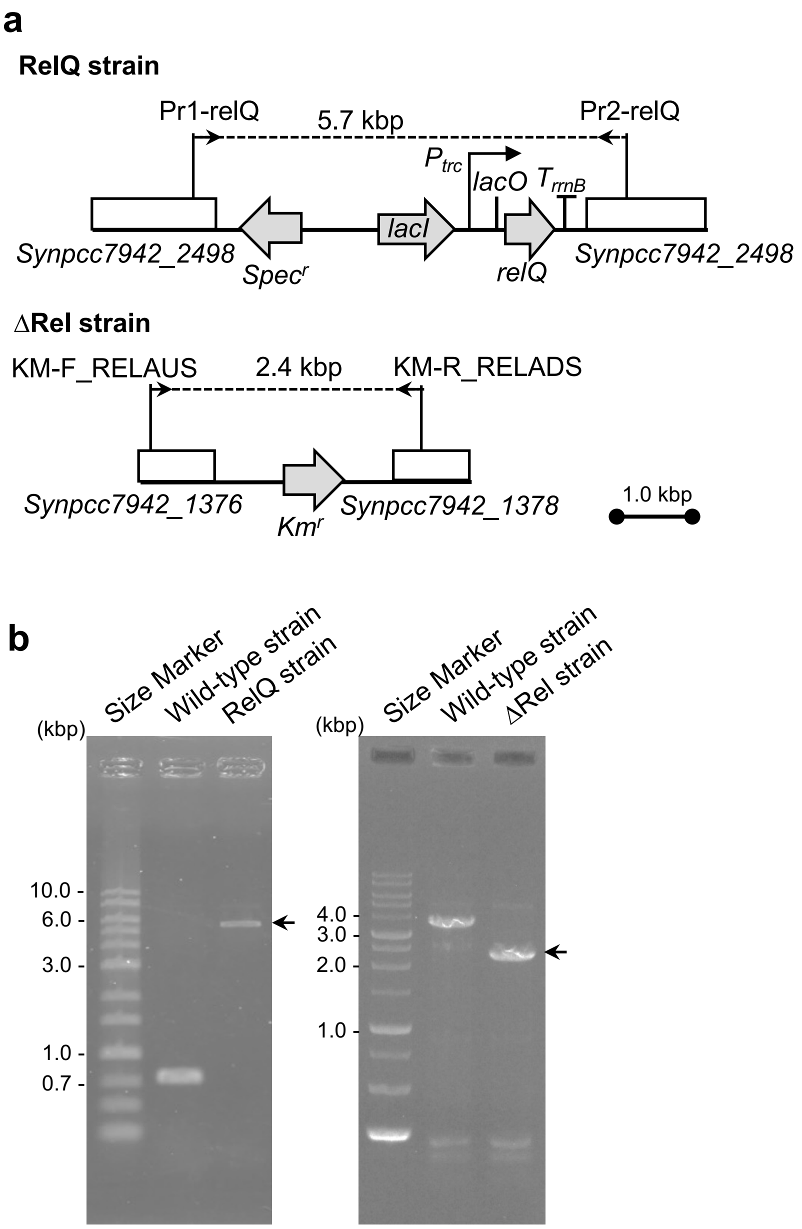

Supplementary Figure 1. Targeted gene integration by homologous recombination. a, The RelQ and ΔRel strains were separately created from the parental strain *S. elongatus* PCC 7942 by homologous recombination. Integration was confirmed by PCR, and the positions of the primer-annealing sites for PCR are indicated by arrows. Abbreviations: *Spec*^{r}, spectinomycin resistance gene; *Km*^{r}, kanamycin resistance gene; P_{trc}, *trc* promoter; *lacI*, lactose repressor gene; *lacO*, *lac* operator; T_{rrnB}, *rrnB* T1 terminator. Locus ID: *Synpcc7942_2498*; *Synpcc7942_1376* and *Synpcc7942_1378*. **b**, PCR analysis of the DNA region containing each gene cassette in the parental strain and each recombinant strain. The sizes of PCR fragments obtained from the corresponding primer pairs (left panel, primer set Pr1-relQ and Pr2-relQ for the fragment containing the *relQ* gene; right panel, primer set KM-F_RELAUS and KM-R_RELADS for the fragment without the *rel* gene) are shown. DNA

size markers are shown as Size Marker.

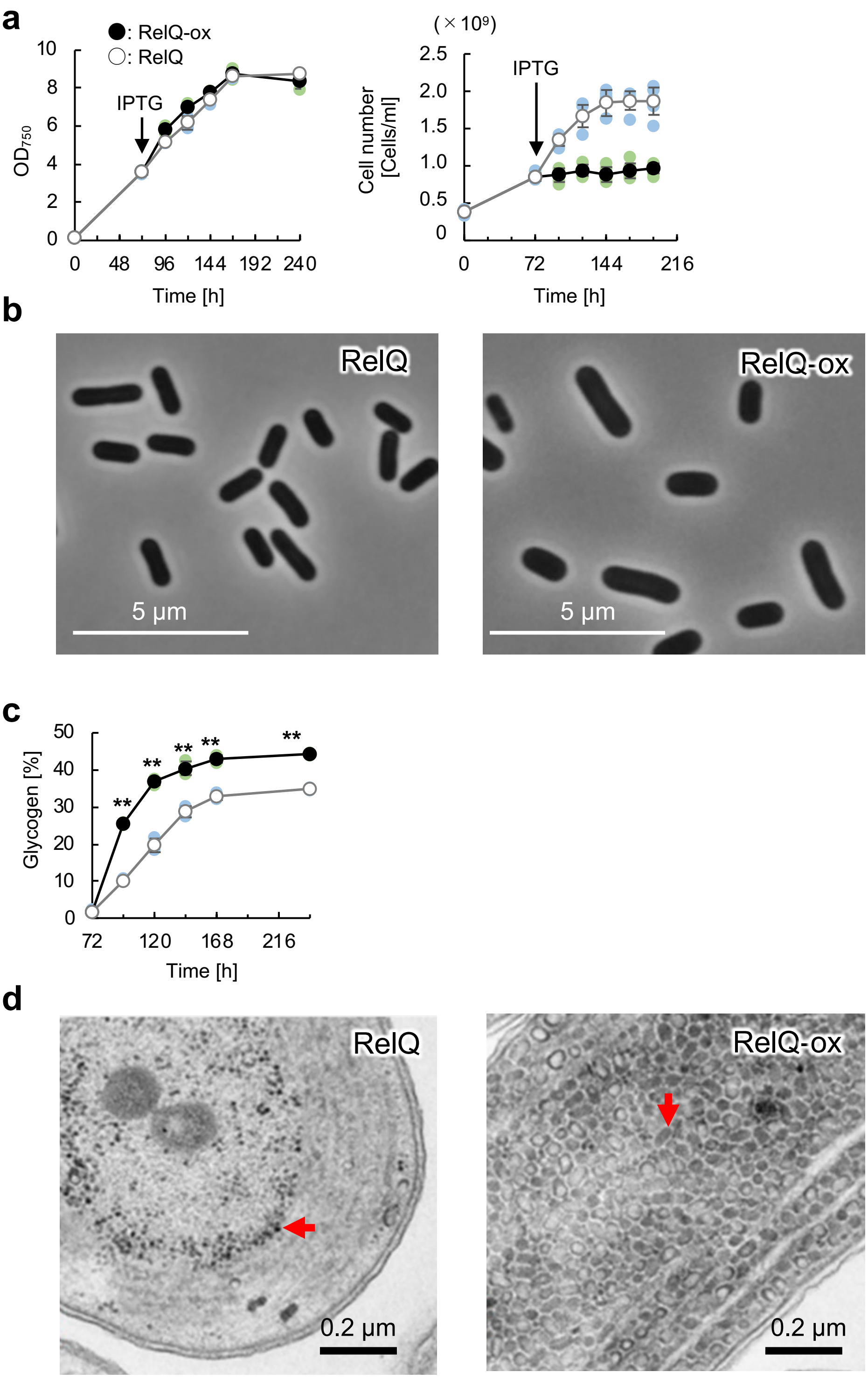

Supplementary Figure 2. The characteristic phenotype of RelQ cells. The RelQ cells were grown for 72 h and then the *relQ* gene was induced; IPTG plus and minus are represented as RelQ-ox and RelQ, respectively. **a**, Growth (cell number and OD_{750}) of RelQ cells with (●, RelQ-ox) or without (○, RelQ) IPTG addition. **b**, The cells were cultivated for 4 days after 72 h of cultivation. The cell size

was observed by optical microscopy. **c**, The intracellular glycogen amounts of RelQ cells with (●, RelQ-ox) or without (○, RelQ) IPTG addition. **d**, The cells were cultivated for 4 days after 72 h of cultivation. The glycogen particle indicated by the red arrowhead were observed by electron microscopy. The bar represents scale. All data are presented as means ± SD (n = 3 independent biological experiments). Statistical significance was determined using Student's *t* test (**P < 0.01).

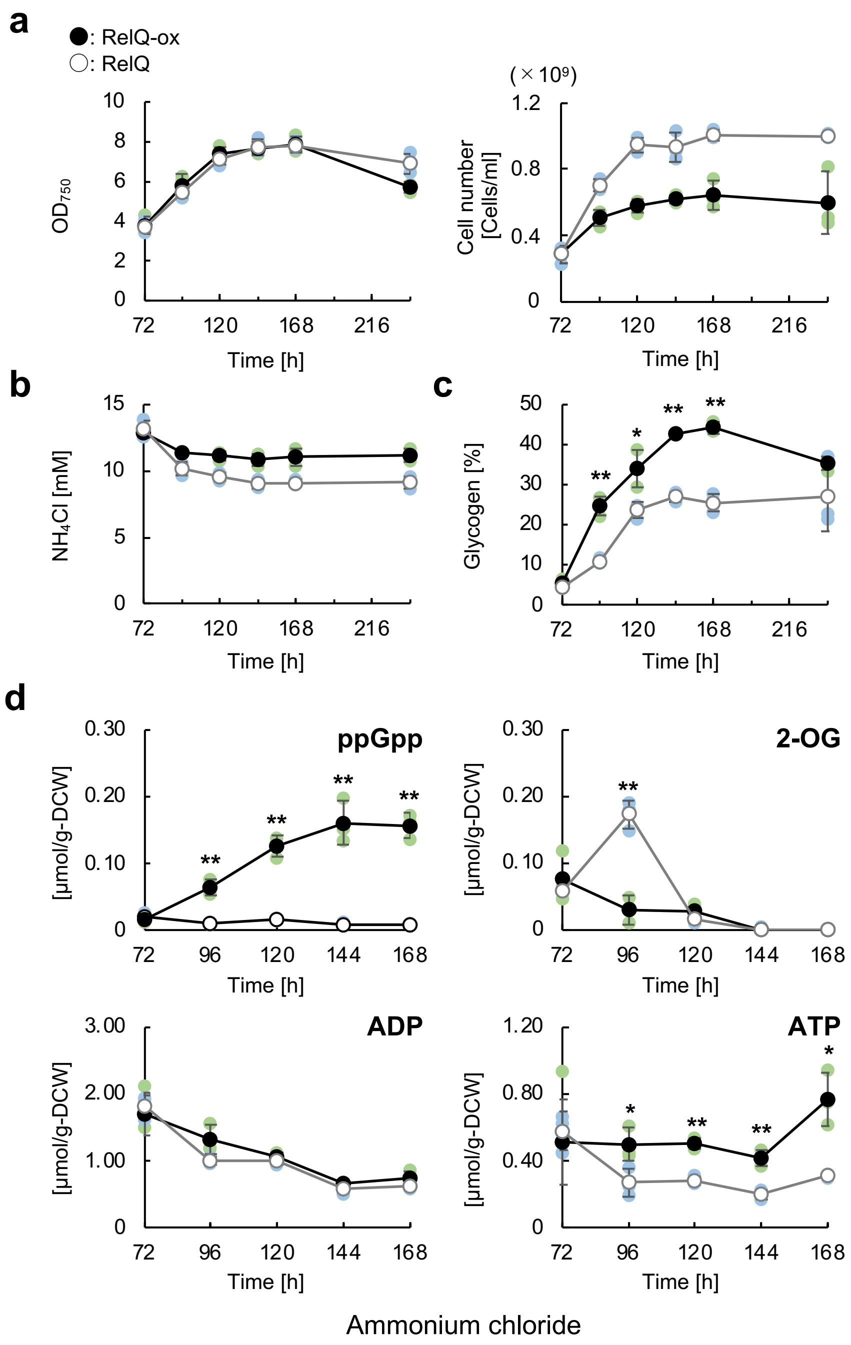

Supplementary Figure 3. ppGpp responses of RelQ cells grown with ammonium chloride. The uptake of ammonium chloride was investigated with ppGpp accumulation, because *S. elongatus* PCC

7942 utilizes a distinct transport system for ammonium ions. The *relQ* gene was induced by the addition of 1 mM IPTG to RelQ cells grown for 72 h in the presence of 17 mM ammonium chloride. Growth (cell number and OD_{750}) (**a**), consumption of NH_4Cl (**b**), and intracellular glycogen amounts (**c**) of RelQ cells with (●, RelQ-ox) or without (○, RelQ) adding IPTG after 72 h. IPTG addition caused the repression of ammonium chloride uptake with an increase of cell number and glycogen accumulation. Intracellular pools of ppGpp, 2-OG, ADP, and ATP were shown (**d**). All data are presented as means ± SD (n = 3 independent biological experiments). Statistical significance was determined using Student's *t* test (**P < 0.01, *P < 0.05).

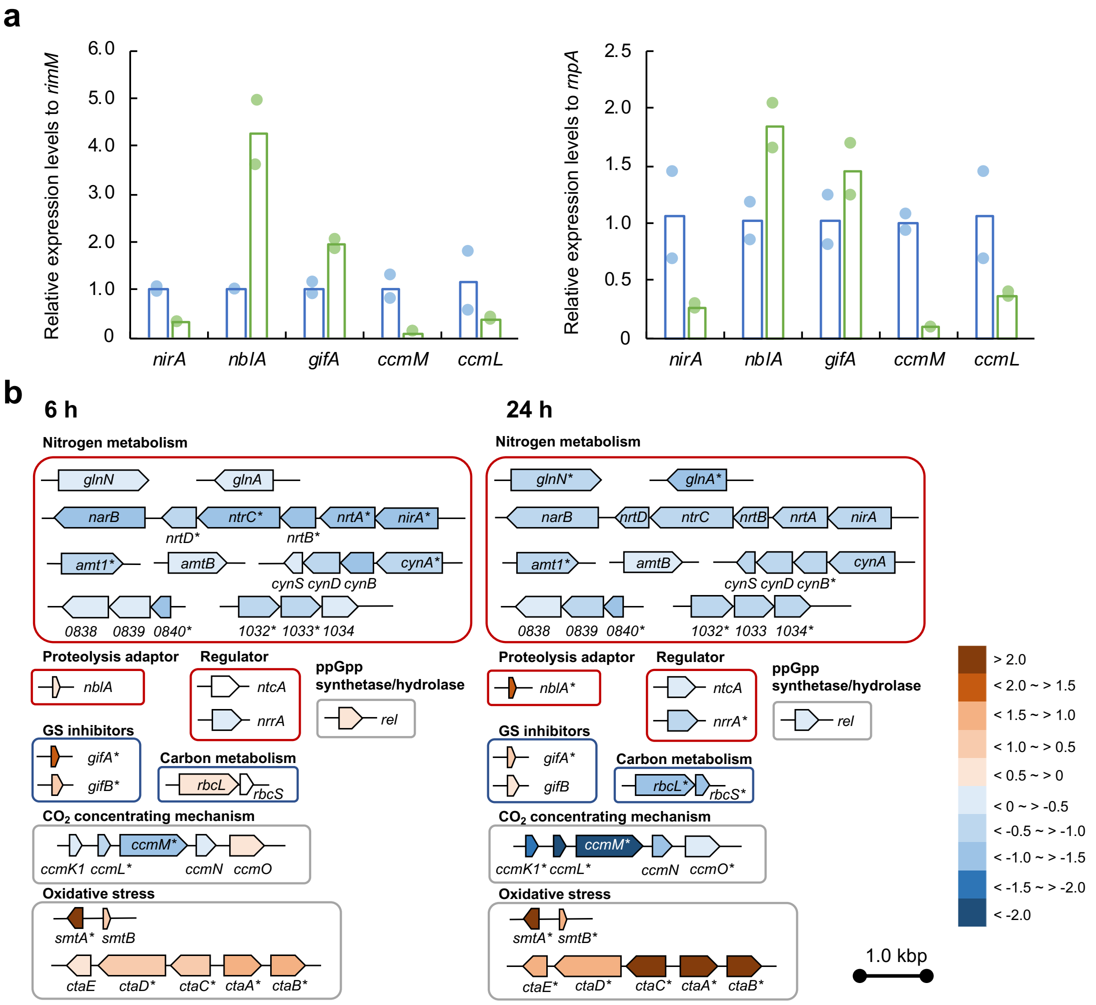

Supplementary Figure 4. Overview of genes with transcript levels significantly changed by ppGpp accumulation. a, Relative expression levels by the comparative Ct method of *nirA*, *nblA*, *gifA*, *ccmL*, and *ccmM* in the RelQ strain. The RelQ cells grown for 72 h were cultivated for 24 h with (green bar) or without adding 1 mM IPTG (blue bar). The primers used for qPCR analysis as follows: nirA-qFw and nirA-qRv for *nirA* transcript; nblA-qFw and nblA-qRv for *nblA* transcript; gifA-qFw and gifA-qRv for *gifA* transcript; ccmL-qFw and ccmL-qRv for *ccmL* transcript; ccmM-qFw and ccmM-qRv for *ccmM* transcript; rnpA-qFw and rnpA-qRv for *rnpA* transcript; rimM-qFw and rimM-

qRv for *rimM* transcript. Relative transcript levels were evaluated using the level of *rnpA* or *rimM* as a reference, and then normalized by the levels of each gene without adding IPTG. All data are presented as means (n = 2 independent biological experiments). **b,** Changes in transcript abundance of representative ORFs, represented by wide orientation arrows, on a $\log_2$ (RelQ-ox/RelQ)-fold change scale, based on transcripts per million (TPM) normalization, are shown as a heat map (left, 6 h; right, 24 h). Genomic organization and predicted transcriptional units of the genes are represented in each panel and functionally categorized. The ORFs under the control of the NtcA-PipX regulon are boxed by red and blue colors, as up- and down-regulated genes, respectively, according to the report by Espinosa et al (29). Gene annotations are shown as follows: *glnN* (Synpcc7942_0169) and *glnA* (Synpcc7942_2156), glutamine synthetase; *narB* (Synpcc7942_1235), nitrate reductase; *nrtABCD* (Synpcc7942_1239 to 1236), nitrite transporter; *nirA* (Synpcc7942_1240), nitrite reductase; *cynABDS* (Synpcc7942_2107 to 2104), cyanate transport and metabolism; *amt1* (Synpcc7942_0442) and *amtB* (Synpcc7942_2279), ammonium transporters; *0838-0840* (Synpcc7942_0838-0840) and *1032-1034* (Synpcc7942_1032-1034), amino acid transport and metabolism; *nblA* (Synpcc7942_2127), phycobilisome degradation protein; *nrrA* (Synpcc7942_2466), nitrogen-regulated response regulator; *ntcA* (Synpcc7942_0127), global nitrogen regulator; *gifA* (Synpcc7942_0900), glutamine synthetase inactivating factor IF7; *gifB* (Synpcc7942_2529), glutamine synthetase inactivating factor IF17; *rbcL* (Synpcc7942_1426) and *rbcS* (Synpcc7942_1427), ribulose 1,5-bisphosphate carboxylase/oxygenases; *ccmK1-ccmLMNO* (Synpcc7942_1421-1425), carboxysome shell proteins; *smtA* (Synpcc7942_1290), metallothionein; *smtB* (Synpcc7942_1291), zinc-responsive repressor; *ctaBACDE* (Synpcc7942_2600-2604), cytochrome c oxidases and maturation; *rel* (Synpcc7942_1377), ppGpp synthetase/hydrolase.

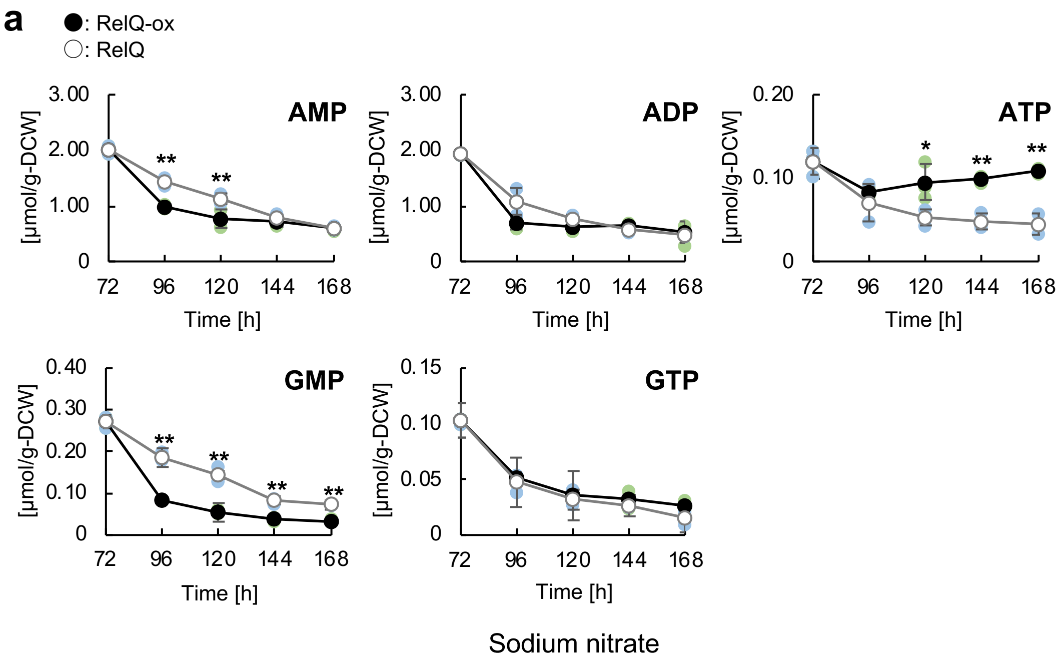

Supplementary Figure 5. Time-course changes of intracellular metabolite pool sizes. The amounts of intracellular nucleic acids (**a**) and amino acids (**b**) and extracellular amino acids (**c**) of the RelQ strain, as described in Figure 1, are shown. The black and broken arrows represent metabolic reaction and pathway, in which metabolic intermediates are formed, respectively. The gray arrow indicates amino acid biosynthetic pathways from each precursor metabolite as follows: Erythrose 4-phosphate (E4P) and PEP for L-tyrosine, L-phenylalanine, and L-tryptophan; 3-PGA for glycine, L-serine, and L-cysteine; Pyruvate for L-valine, L-leucine, and L-alanine; Oxaloacetate (OXA) for L-aspartate, L-asparagine, L-lysine, L-methionine, L-isoleucine, and L-threonine; and 2-oxoglutarate (2-OG) for L-glutamate, L-glutamine, L-arginine, L-histidine, and L-proline. Statistical significance between each sampling point of RelQ-ox and RelQ was determined using Student's *t* test (**P < 0.01, *P < 0.05).

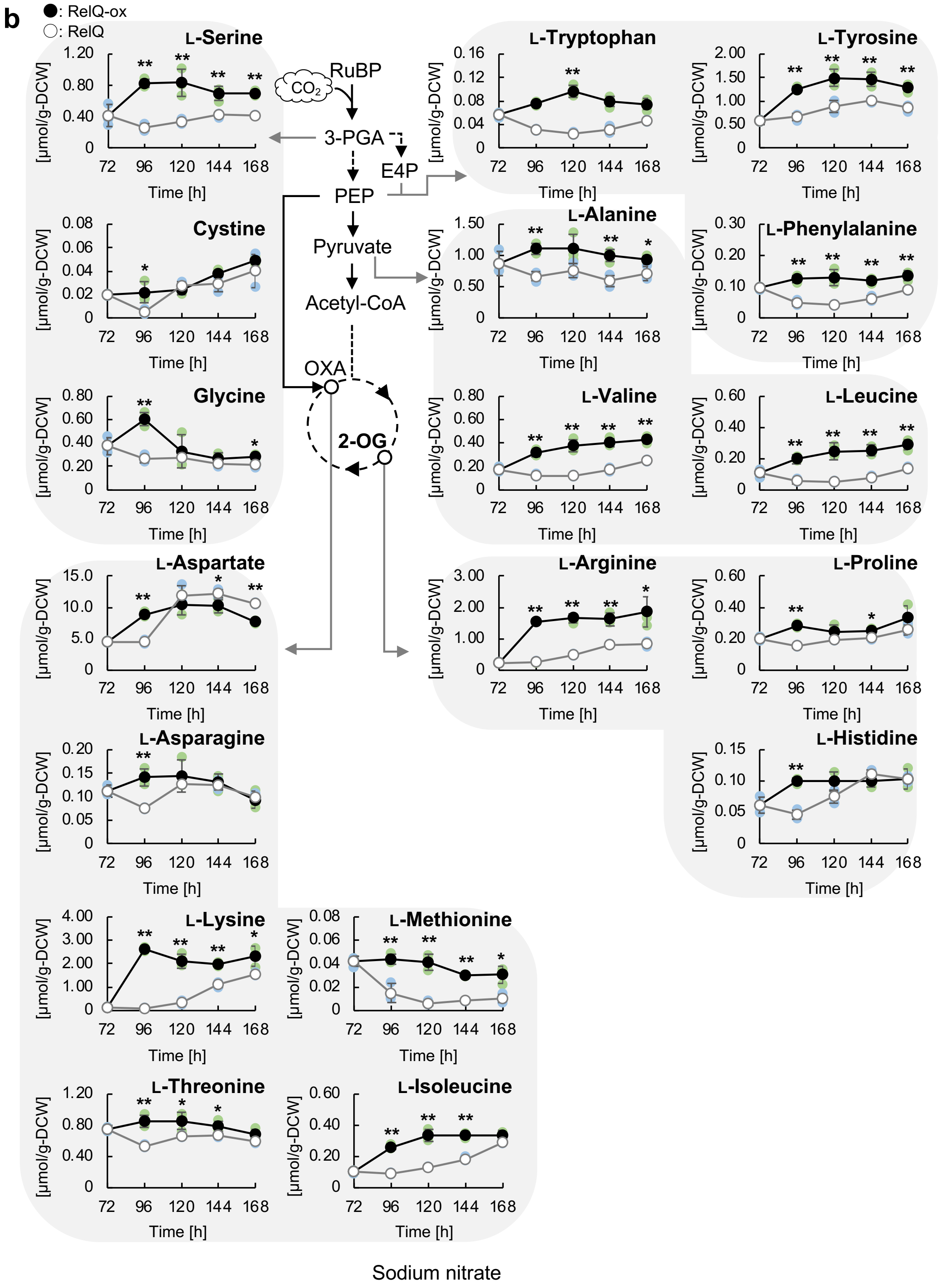

Supplementary Figure 5. (Continued)

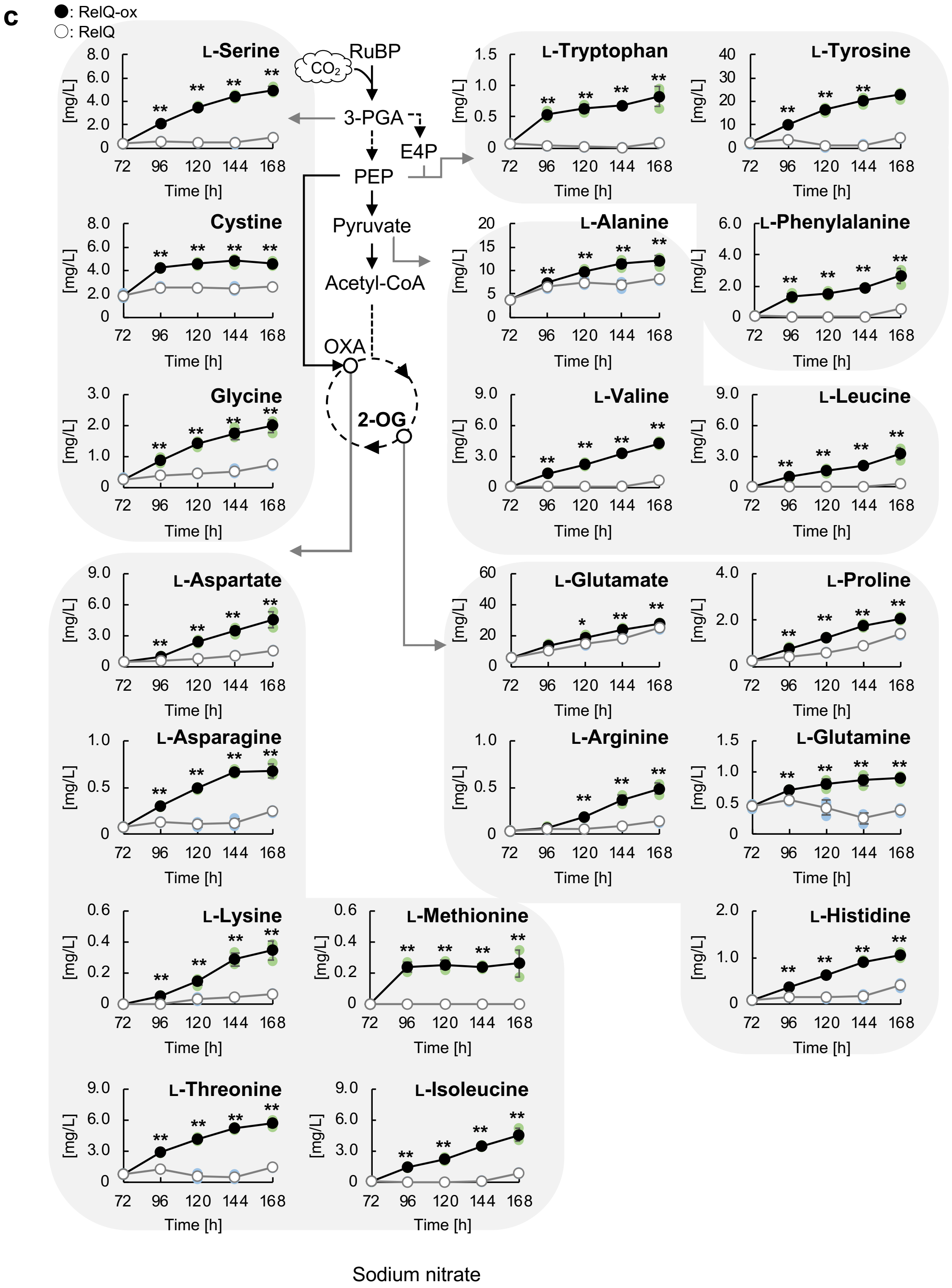

Supplementary Figure 5. (Continued)

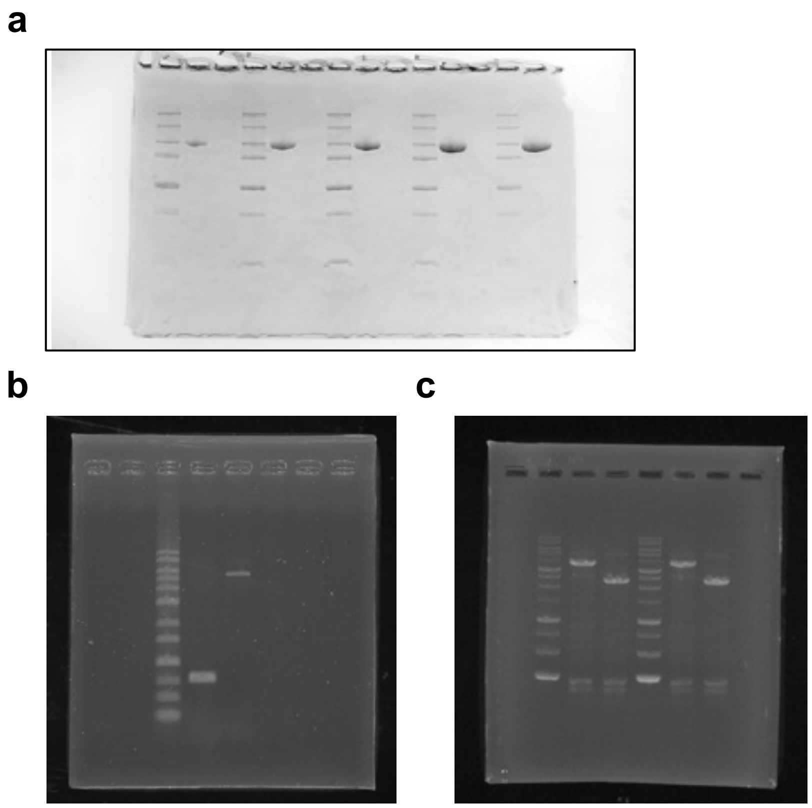

Supplementary Figure 6. Unedited images for the gels. The unedited image for SDS-PAGE gel of purified recombinant Synpcc7942_0903 proteins presented in Figure 4b (**a**). The unedited images for agarose gels of PCR products containing the *relQ* gene cassette (**b**) and without the *rel* gene (**c**) presented in Supplemental Figure 1b.

Supplementary Table 1. Primers used in this study.

Primer	Sequence (5′ to 3′)
RelQ-Fw	TTCGAGCTCCACCGCATGGATGACAAACAATGGGA
RelQ-Rv	TGCCTGCAGGTCGACCTATTGTTGCTCGCTTCCT
pNSHA-Fw	GTCGACCTGCAGGCATGC
pNSHA-Rv	GGATCCTGCATAGTCCGGGAC
RELA-US-F	CGCCTACACCTGCATTGTTGG
RELA-US-R_KM	CAATTCCACACGAACGTGCGATCGCTCC
KM-F_RELAUS	CGCACGTTCGAATTCCCCGGATCCGTCGA
KM-R_RELADS	GATAGCTGAGTGTTAGAAAAACTCATCGAGCA
RELA-DS-F_KM	GTTTTTCTAACACTCAGCTATCCAACTGATGC
RELA-DS-R	GCGATCGCGGCTCAGC
Synpcc7942_0903-Fw	GCCGCGCGGCAGCCATATGTTGGAAGCCTACCGC
Synpcc7942_0903-Rv	CTCGAGTGCGGCCGCAAGCTACACAGTGGTCAAAATGC
Pr1-relQ	ATGCCCAGGCTGCCTCTG
Pr2-relQ	ACCCCGCTGAACGCCATC
nirA-qFw	ATTGCATGCAGTGGGCTTAACC
nirA-qRV	TGCGCGGCAAATTGGTGAATTC
nblA-qFw	TTCCCTCAGTGTTGAACAGCAG
nblA-qRV	TGTTCTCGTGTGCCATCTTCTG
gifA-qFw	AATCGCGTGAACTGGTGATGCG
gifA-qRV	TGCCGTTGTAGTGCCGCATTTG
ccmL-qFw	ATGTGCGCGATTGTCAGGAACG
ccmL-qRV	TTAGCTGTGCTCGCGTTTGTCG
ccmM-qFw	AATCATGATGCACGCCCTTGTC
ccmM-qRV	AATGATGTGGCGGGCAAATTCC
rnpA-qFw	TCACTTTTGTTGCGCTGGTTGC
rnpA-qRv	AAAACTGACTCGTGCTGGTGTCG
rimM-qFw	AACAACCATCGAAACCGCTTGGC
rimM-qRv	AACTCACAACCGCGCAATGCTTC
