# Supplementary material for: ppGpp accumulation reduces the expression of the global nitrogen homeostasis-modulating NtcA regulon by affecting 2-oxoglutarate levels
Source: Commun Biol. 2023 Dec 25;6:1285. doi: 10.1038/s42003-023-05632-1 (PMC10749895; doi:10.1038/s42003-023-05632-1)
Supplement: Supplementary file 3 — Description of Additional Supplementary Files [file 42003_2023_5632_MOESM3_ESM.pdf]

Description of Additional Supplementary Files

File name: Supplementary Data 1.
Description: The source data behind the graphs in this study.

File name: Supplementary Data 2.
Description: DNA sequences of genes used in this study.

File name: Supplementary Data 3.
Description: mRNA expression data for all genes with ordered locus names.
