# Supplementary material for: ppGpp accumulation reduces the expression of the global nitrogen homeostasis-modulating NtcA regulon by affecting 2-oxoglutarate levels
Source: Commun Biol. 2023 Dec 25;6:1285. doi: 10.1038/s42003-023-05632-1 (PMC10749895; doi:10.1038/s42003-023-05632-1)
Supplement: Supplementary file 5 — Supplementary Data 2 [file 42003_2023_5632_MOESM5_ESM.docx]

Supplementary Data 2

DNA sequences of genes used in this study.

> pNSI-RelQ

CCCATTCGCCATTCAGGCTGCGCAACTGTTGGGAAGGGCGATCGGTGCGGGCCTCTTCGCTATTACGCCAGCTGGCGAAAGGGGGATGTGCTGCAAGGCGATTAAGTTGGGTAACGCCAGGGTTTTCCCAGTCACGACGTTGTAAAACGACGGCCAGTGCCAAGCTAAAAGCGCTCCGCATGGATCTGACCAACATGATCATTGAGTTGCGCGTTTCCAATGCCTTCTCCAAGGGCGGCATTCCCCTGACTGTTGAAGGCGTTGCCAATATCAAGATTGCTGGGGAAGAACCGACCATCCACAACGCGATCGAGCGGCTGCTTGGCAAAAACCGTAAGGAAATCGAGCAAATTGCCAAGGAGACCCTCGAAGGCAACTTGCGTGGTGTTTTAGCCAGCCTCACGCCGGAGCAGATCAACGAGGACAAAATTGCCTTTGCCAAAAGTCTGCTGGAAGAGGCGGAGGATGACCTTGAGCAGCTGGGTCTAGTCCTCGATACGCTGCAAGTCCAGAACATTTCCGATGAGGTCGGTTATCTCTCGGCTAGTGGACGCAAGCAGCGGGCTGATCTGCAGCGAGATGCCCGAATTGCTGAAGCCGATGCCCAGGCTGCCTCTGCGATCCAAACGGCCGAAAATGACAAGATCACGGCCCTGCGTCGGATCGATCGCGATGTAGCGATCGCCCAAGCCGAGGCCGAGCGCCGGATTCAGGATGCGTTGACGCGGCGCGAAGCGGTGGTGGCCGAAGCTGAAGCGGACATTGCTACCGAAGTCGCTCGTAGCCAAGCAGAACTCCCTGTGCAGCAGGAGCGGATCAAACAGGTGCAGCAGCAACTTCAAGCCGATGTGATCGCCCCAGCTGAGGCAGCTTGTAAACGGGCGATCGCGGAAGCGCGGGGGGCCGCCGCCCGTATCGTCGAAGATGGAAAAGCTCAAGCGGAAGGGACCCAACGGCTGGCGGAGGCTTGGCAGACCGCTGGTGCTAATGCCCGCGACATCTTCCTGCTCCAGAAGCTCGACTATGCTTGTAAACCGTTTTGTGAAAAAATTTTTAAAATAAAAAAGGGGACCTCTAGGGTCCCCAATTAATTAGTAATATAATCTATTAAAGGTCATTCAAAAGGTCATCCACCGGATCAGCTTAGTAAAGCCCTCGCTAGATTTTAATGCGGATGTTGCGATTACTTCGCCAACTATTGCGATAACAAGAAAAAGCCAGCCTTTCATGATATATCTCCCAATTTGTGTAGGGCTTATTATGCACGCTTAAAAATAATAAAAGCAGACTTGACCTGATAGTTTGGCTGTGAGCAATTATGTGCTTAGTGCATCTAACGCTTGAGTTAAGCCGCGCCGCGAAGCGGCGTCGGCTTGAACGAATTGTTAGACATTATTTGCCGACTACCTTGGTGATCTCGCCTTTCACGTAGTGGACAAATTCTTCCAACTGATCTGCGCGCGAGGCCAAGCGATCTTCTTCTTGTCCAAGATAAGCCTGTCTAGCTTCAAGTATGACGGGCTGATACTGGGCCGGCAGGCGCTCCATTGCCCAGTCGGCAGCGACATCCTTCGGCGCGATTTTGCCGGTTACTGCGCTGTACCAAATGCGGGACAACGTAAGCACTACATTTCGCTCATCGCCAGCCCAGTCGGGCGGCGAGTTCCATAGCGTTAAGGTTTCATTTAGCGCCTCAAATAGATCCTGTTCAGGAACCGGATCAAAGAGTTCCTCCGCCGCTGGACCTACCAAGGCAACGCTATGTTCTCTTGCTTTTGTCAGCAAGATAGCCAGATCAATGTCGATCGTGGCTGGCTCGAAGATACCTGCAAGAATGTCATTGCGCTGCCATTCTCCAAATTGCAGTTCGCGCTTAGCTGGATAACGCCACGGAATGATGTCGTCGTGCACAACAATGGTGACTTCTACAGCGCGGAGAATCTCGCTCTCTCCAGGGGAAGCCGAAGTTTCCAAAAGGTCGTTGATCAAAGCTCGCCGCGTTGTTTCATCAAGCCTTACGGTCACCGTAACCAGCAAATCAATATCACTGTGTGGCTTCAGGCCGCCATCCACTGCGGAGCCGTACAAATGTACGGCCAGCAACGTCGGTTCGAGATGGCGCTCGATGACGCCAACTACCTCTGATAGTTGAGTCGATACTTCGGCGATCACCGCTTCCCTCATGATGTTTAACTTTGTTTTAGGGCGACTGCCCTGCTGCGTAACATCGTTGCTGCTCCATAACATCAAACATCGACCCACGGCGTAACGCGCTTGCTGCTTGGATGCCCGAGGCATAGACTGTACCCCAAAAAAACAGTCATAACAAGCCATGAAAACCGCCACTGCGCCGTTACCACCGCTGCGTTCGGTCAAGGTTCTGGACCAGTTGCGTGAGCGCATACGCTACTTGCATTACAGCTTACGAACCGAACAGGCTTATGTCCACTGGGTTCGTGCCTTCATCCGTTTCCACGGTGTGCGTCACCCGGCAACCTTGGGCAGCAGCGAAGTCGAGGCATTTCTGTCCTGGCTGGCGAACGAGCGCAAGGTTTCGGTCTCCACGCATCGTCAGGCATTGGCGGCCTTGCTGTTCTTCTACGGCAAGGTGCTGTGCACGGATCTGCCCTGGCTTCAGGAGATCGGAAGACCTCGGCCGTCGCGGCGCTTGCCGGTGGTGCTGACCCCGGATGAAGTGGTTCGCATCCTCGGTTTTCTGGAAGGCGAGCATCGTTTGTTCGCCCAGCTTCTGTATGGAACGGGCATGCGGATCAGTGAGGGTTTGCAACTGCGGGTCAAGGATCTGGATTTCGATCACGGCACGATCATCGTGCGGGAGGGCAAGGGCTCCAAGGATCGGGCCTTGATGTTACCCGAGAGCTTGGCACCCAGCCTGCGCGAGCAGGGGAATTGATCCGGTGGATGACCTTTTGAATGACCTTTAATAGATTATATTACTAATTAATTGGGGACCCTAGAGGTCCCCTTTTTTATTTTAAAAATTTTTTCACAAAACGGTTTACAAGCATAGTCGAGTTACGTTGACACCATCGAATGGTGCAAAACCTTTCGCGGTATGGCATGATAGCGCCCGGAAGAGAGTCAATTCAGGGTGGTGAATGTGAAACCAGTAACGTTATACGATGTCGCAGAGTATGCCGGTGTCTCTTATCAGACCGTTTCCCGCGTGGTGAACCAGGCCAGCCACGTTTCTGCGAAAACGCGGGAAAAAGTGGAAGCGGCGATGGCGGAGCTGAATTACATTCCCAACCGCGTGGCACAACAACTGGCGGGCAAACAGTCGTTGCTGATTGGCGTTGCCACCTCCAGTCTGGCCCTGCACGCGCCGTCGCAAATTGTCGCGGCGATTAAATCTCGCGCCGATCAACTGGGTGCCAGCGTGGTGGTGTCGATGGTAGAACGAAGCGGCGTCGAAGCCTGTAAAGCGGCGGTGCACAATCTTCTCGCGCAACGCGTCAGTGGGCTGATCATTAACTATCCGCTGGATGACCAGGATGCCATTGCTGTGGAAGCTGCCTGCACTAATGTTCCGGCGTTATTTCTTGATGTCTCTGACCAGACACCCATCAACAGTATTATTTTCTCCCATGAAGACGGTACGCGACTGGGCGTGGAGCATCTGGTCGCATTGGGTCACCAGCAAATCGCGCTGTTAGCGGGCCCATTAAGTTCTGTCTCGGCGCGTCTGCGTCTGGCTGGCTGGCATAAATATCTCACTCGCAATCAAATTCAGCCGATAGCGGAACGGGAAGGCGACTGGAGTGCCATGTCCGGTTTTCAACAAACCATGCAAATGCTGAATGAGGGCATCGTTCCCACTGCGATGCTGGTTGCCAACGATCAGATGGCGCTGGGCGCAATGCGCGCCATTACCGAGTCCGGGCTGCGCGTTGGTGCGGATATCTCGGTAGTGGGATACGACGATACCGAAGACAGCTCATGTTATATCCCGCCGTTAACCACCATCAAACAGGATTTTCGCCTGCTGGGGCAAACCAGCGTGGACCGCTTGCTGCAACTCTCTCAGGGCCAGGCGGTGAAGGGCAATCAGCTGTTGCCCGTCTCACTGGTGAAAAGAAAAACCACCCTGGCGCCCAATACGCAAACCGCCTCTCCCCGCGCGTTGGCCGATTCATTAATGCAGCTGGCACGACAGGTTTCCCGACTGGAAAGCGGGCAGTGAGCGCAACGCAATTAATGTGAGTTAGCGCGAATTGATCTGGTTTGACAGCTTATCATCGACTGCACGGTGCACCAATGCTTCTGGCGTCAGGCAGCCATCGGAAGCTGTGGTATGGCTGTGCAGGTCGTAAATCACTGCATAATTCGTGTCGCTCAAGGCGCACTCCCGTTCTGGATAATGTTTTTTGCGCCGACATCATAACGGTTCTGGCAAATATTCTGAAATGAGCTGTTGACAATTAATCATCCGGCTCGTATAATGTGTGGAATTGTGAGCGGATAACAATTTCACACAGGAAACAGACCATGGAATTCgagctccaccgcATGGATGACAAACAATGGGAGCGTTTTTTAGTGCCGTACCGCCAGGCTGTCGAAGAGTTGAAAGTGAAGCTCAAGGGGATCCGCACACTATATGAATACGAGGACGACCATTCACCGATCGAATTTGTGACCGGACGCGTCAAGCCTGTGGCGAGCATACTTGAAAAAGCGAGACGGAAAAGCATACCGCTGCATGAAATTGAAACCATGCAGGACATTGCTGGCCTTAGAATCATGTGCCAATTTGTCGATGACATCCAAATCGTAAAAGAGATGCTTTTTGCCAGAAAAGATTTCACCGTTGTGGATCAGAGGGATTATATTGCGGAACATAAAGAGAGCGGATACCGATCATACCATCTTGTTGTGCTGTACCCTTTGCAGACGGTATCCGGAGAAAAGCATGTTCTCGTAGAAATACAGATCCGTACACTGGCGATGAATTTTTGGGCGACCATTGAACATTCATTGAATTATAAATACAGCGGAAACATTCCTGAAAAAGTAAAACTAAGGCTTCAGCGCGCTTCTGAAGCGGCTTCCCGGCTTGATGAAGAGATGTCGGAGATCAGGGGTGAAGTACAGGAAGCTCAAGCTGCCTTTTCAAGAAAGAAAAAAGGAAGCGAGCAACAATAGGTCGACCTGCAGGCATGCAAGCTTGGCTGTTTTGGCGGATGAGAGAAGATTTTCAGCCTGATACAGATTAAATCAGAACGCAGAAGCGGTCTGATAAAACAGAATTTGCCTGGCGGCAGTAGCGCGGTGGTCCCACCTGACCCCATGCCGAACTCAGAAGTGAAACGCCGTAGCGCCGATGGTAGTGTGGGGTCTCCCCATGCGAGAGTAGGGAACTGCCAGGCATCAAATAAAACGAAAGGCTCAGTCGAAAGACTGGGCCTTTCGTTTTATCTGTTGTTTGTCGGTGAACGCTCTCCTGAGTAGGACAAATCCGCCGGGAGCGGATTTGAACGTTGCGAAGCAACGGCCCGGAGGGTGGCGGGCAGGACGCCCGCCATAAACTGCCAGGCATCAAATTAAGCAGAAGGCCATCCTGACGGATGGCCTTTTTGCGTTTCTACAAACTCTTTTTGTTTATTTTTCTAAATACATTCAAATATGTATCCGCTCATGAGACAATAACCCTGATAAATGCTTCAATAATATTGAAAAAGGAAGAGTATGAGTATTCAACATTTCCGTGTCGCCCTTATTCCCTTTTTTGCGGCATTTTGCCTTCCTGTTTTTGCTCACCCAGAAACGCTGGTGAAAGTAAAAGATGCTGAAGATCAGTTGGGTGCACGAGTGGGTTACATCGAACTGGATCTCAACAGCGGTAAGATCCTTGAGAGTTTTCGCCCCGAAGAACGTTTTCCAATGATGAGCACTTTTAAAGTTCTGCTATGTGGCGCGGTATTATCCCGTGTTGACGCCGGGCAAGAGCAACTCGGTCGCCGCATACACTATTCTCAGAATGACTTGGTTGAGTATCGACGTGGAGTCGATCACTGTGATTGGCGAAGGGGAAGGCAGCGCTACCCAAATCGCTAGCTTGCTGGAGAAGCTGAAACAAACCACGGGCATTGATCTGGCGAAATCCCTACCGGGTCAATCCGACTCGCCCGCTGCGAAGTCCTAAGAGATAGCGATGTGACCGCGATCGCTTGTCAAGAATCCCAGTGATCCCGAACCATAGGAAGGCAAGCTCAATGCTTGCCTCGTCTTGAGGACTATCTAGATGTCTGTGGAACGCACATTTATTGCCATCAAGCCCGATGGCGTTCAGCGGGGTTTGGTCGGTACGATCATCGGCCGCTTTGAGCAAAAAGGCTTCAAACTGGTGGGCCTAAAGCAGCTGAAGCCCAGTCGCGAGCTGGCCGAACAGCACTATGCTGTCCACCGCGAGCGCCCCTTCTTCAATGGCCTCGTCGAGTTCATCACCTCTGGGCCGATCGTGGCGATCGTCTTGGAAGGCGAAGGCGTTGTGGCGGCTGCTCGCAAGTTGATCGGCGCTACCAATCCGCTGACGGCAGAACCGGGCACCATCCGTGGTGATTTTGGTGTCAATATTGGCCGCAACATCATCCATGGCTCGGATGCAATCGAAACAGCACAACAGGAAATTGCTCTCTGGTTTAGCCCAGCAGAGCTAAGTGATTGGACCCCCACGATTCAACCCTGGCTGTACGAATAAGGTCTGCATTCCTTCAGAGAGACATTGCCATGCCCGTGCTGCGATCGCCCTTCCAAGCTGCCTTGCCCCGCTGTTTCGGGCTGGCAGCCCTGGCGTTGGGGCTGGCGACCGCTTGCCAAGAAAGCAGCGCTCCAATTCCCTATAGTGAGTCGTATTAAATTCGTAATCATGGTCATAGCTGTTTCCTGTGTGAAATTGTTATCCGCTCACAATTCCACACAACATACGAGCCGGAAGCATAAAGTGTAAAGCCTGGGGTGCCTAATGAGTGAGCTAACTCACATTAATTGCGTTGCGCTCACTGCCCGCTTTCCAGTCGGGAAACCTGTCGTGCCAGCTGCATTAATGAATCGGCCAACGCGCGGGGAGAGGCGGTTTGCGTATTGGGCGCTCTTCCGCTTCCTCGCTCACTGACTCGCTGCGCTCGGTCGTTCGGCTGCGGCGAGCGGTATCAGCTCACTCAAAGGCGGTAATACGGTTATCCACAGAATCAGGGGATAACGCAGGAAAGAACATGTGAGCAAAAGGCCAGCAAAAGGCCAGGAACCGTAAAAAGGCCGCGTTGCTGGCGTTTTTCCATAGGCTCCGCCCCCCTGACGAGCATCACAAAAATCGACGCTCAAGTCAGAGGTGGCGAAACCCGACAGGACTATAAAGATACCAGGCGTTTCCCCCTGGAAGCTCCCTCGTGCGCTCTCCTGTTCCGACCCTGCCGCTTACCGGATACCTGTCCGCCTTTCTCCCTTCGGGAAGCGTGGCGCTTTCTCATAGCTCACGCTGTAGGTATCTCAGTTCGGTGTAGGTCGTTCGCTCCAAGCTGGGCTGTGTGCACGAACCCCCCGTTCAGCCCGACCGCTGCGCCTTATCCGGTAACTATCGTCTTGAGTCCAACCCGGTAAGACACGACTTATCGCCACTGGCAGCAGCCACTGGTAACAGGATTAGCAGAGCGAGGTATGTAGGCGGTGCTACAGAGTTCTTGAAGTGGTGGCCTAACTACGGCTACACTAGAAGGACAGTATTTGGTATCTGCGCTCTGCTGAAGCCAGTTACCTTCGGAAAAAGAGTTGGTAGCTCTTGATCCGGCAAACAAACCACCGCTGGTAGCGGTGGTTTTTTTGTTTGCAAGCAGCAGATTACGCGCAGAAAAAAAGGATCTCAAGAAGATCCTTTGATCTTTTCTACGGGGTCTGACGCTCAGTGGAACGAAAACTCACGTTAAGGGATTTTGGTCATGAGATTATCAAAAAGGATCTTCACCTAGATCCTTTTAAATTAAAAATGAAGTTTTAAATCAATCTAAAGTATATATGAGTAAACTTGGTCTGACAGTTACCAATGCTTAATCAGTGAGGCACCTATCTCAGCGATCTGTCTATTTCGTTCATCCATAGTTGCCTGACTCCCCGTCGTGTAGATAACTACGATACGGGAGGGCTTACCATCTGGCCCCAGTGCTGCAATGATACCGCGAGACCCACGCTCACCGGCTCCAGATTTATCAGCAATAAACCAGCCAGCCGGAAGGGCCGAGCGCAGAAGTGGTCCTGCAACTTTATCCGCCTCCATCCAGTCTATTAATTGTTGCCGGGAAGCTAGAGTAAGTAGTTCGCCAGTTAATAGTTTGCGCAACGTTGTTGCCATTGCTACAGGCATCGTGGTGTCACGCTCGTCGTTTGGTATGGCTTCATTCAGCTCCGGTTCCCAACGATCAAGGCGAGTTACATGATCCCCCATGTTGTGCAAAAAAGCGGTTAGCTCCTTCGGTCCTCCGATCGTTGTCAGAAGTAAGTTGGCCGCAGTGTTATCACTCATGGTTATGGCAGCACTGCATAATTCTCTTACTGTCATGCCATCCGTAAGATGCTTTTCTGTGACTGGTGAGTACTCAACCAAGTCATTCTGAGAATAGTGTATGCGGCGACCGAGTTGCTCTTGCCCGGCGTCAATACGGGATAATACCGCGCCACATAGCAGAACTTTAAAAGTGCTCATCATTGGAAAACGTTCTTCGGGGCGAAAACTCTCAAGGATCTTACCGCTGTTGAGATCCAGTTCGATGTAACCCACTCGTGCACCCAACTGATCTTCAGCATCTTTTACTTTCACCAGCGTTTCTGGGTGAGCAAAAACAGGAAGGCAAAATGCCGCAAAAAAGGGAATAAGGGCGACACGGAAATGTTGAATACTCATACTCTTCCTTTTTCAATATTATTGAAGCATTTATCAGGGTTATTGTCTCATGAGCGGATACATATTTGAATGTATTTAGAAAAATAAACAAATAGGGGTTCCGCGCACATTTCCCCGAAAAGTGCCACCTGACGCGCCCTGTAGCGGCGCATTAAGCGCGGCGGGTGTGGTGGTTACGCGCAGCGTGACCGCTACACTTGCCAGCGCCCTAGCGCCCGCTCCTTTCGCTTTCTTCCCTTCCTTTCTCGCCACGTTCGCCGGCTTTCCCCGTCAAGCTCTAAATCGGGGCATCCCTTTAGGGTTCCGATTTAGTGCTTTACGGCACCTCGACCCCAAAAAACTTGATTAGGGTGATGGTTCACGTAGTGGGCCATCGCCCTGATAGACGGTTTTTCGCCCTTTGACGTTGGAGTCCACGTTCTTTAATAGTGGACTCTTGTTCCAAACTGGAACAACACTCAACCCTATCTCGGTCTATTCTTTTGATTTATAAGGGATTTTGCCGATTTCGGCCTATTGGTTAAAAAATGAGCTGATTTAACAAAAATTTAACGCGAATTTTAACAAAATATTAACAAAATATTAACGTTTACAATTT

> PCR fragment without rel gene

CGCCTACACCTGCATTGTTGGCAACACGACCGCCATCAGCCGTAGGCGGGCCGGTGATGACCTCTGAAATGGCATAGATCGGTACTGAGACTCCATTGAGGACAAGCGCTTGTGCAACAAACCGACTGCCCTGCTGATTAGTTTCAAAGCGGCCGAGCACTGGCGTCCCGATCGGTAGGATGACCTGGCCCCTGCTATCCGTGACATTTTGGTCAACAGTCATGACCTCTTGAATTGGTAGAGCGCTTTGCAAGCTCAGACCAGGACCTGTGTAGCGGAGCCGCACCACAAACCGATCGCTCAAGGGTTTCAAGGGGGTTGTGCTGAGTGACGAGCCACCGCCAGCCGGCACGGTCGGCAGTGGAATACTGGACTGGGCCCAAGCGGTCGAAGCTGACAGTGCGGCTAACACGCCAGCGATCGCGACTGCAGATTTGCGCATCATTTCCATCCATCGCCTTTCGCACAGCTAGCTTGCCATTTTCCCACCAGGAATCAAGACCTAGGTTTGCCGTTTGCACCAAGAATGACTGGATTCAGACCCAGAATCTTGCGATTCAGTCCACTAGCATTCCTGTCACCCCCCTGACTCACAGGAAATTAACGGTTAAGATCGGTGCACAACTGCCGAGCTTCCCAATGAACTGCTGCAGTTGCACCAGCCAAAAGGGGAGCGATCGCACGTCGCACGTTCGAATTCCCCGGATCCGTCGACCTGCAGGGGGGGGGGGGAAAGCCACGTTGTGTCTCAAAATCTCTGATGTTACATTGCACAAGATAAAAATATATCATCATGAACAATAAAACTGTCTGCTTACATAAACAGTAATACAAGGGGTGTTATGAGCCATATTCAACGGGAAACGTCTTGCTCGAGGCCGCGATTAAATTCCAACATGGATGCTGATTTATATGGGTATAAATGGGCTCGCGATAATGTCGGGCAATCAGGTGCGACAATCTATCGATTGTATGGGAAGCCCGATGCGCCAGAGTTGTTTCTGAAACATGGCAAAGGTAGCGTTGCCAATGATGTTACAGATGAGATGGTCAGACTAAACTGGCTGACGGAATTTATGCCTCTTCCGACCATCAAGCATTTTATCCGTACTCCTGATGATGCATGGTTACTCACCACTGCGATCCCCGGGAAAACAGCATTCCAGGTATTAGAAGAATATCCTGATTCAGGTGAAAATATTGTTGATGCGCTGGCAGTGTTCCTGCGCCGGTTGCATTCGATTCCTGTTTGTAATTGTCCTTTTAACAGCGATCGCGTATTTCGTCTCGCTCAGGCGCAATCACGAATGAATAACGGTTTGGTTGATGCGAGTGATTTTGATGACGAGCGTAATGGCTGGCCTGTTGAACAAGTCTGGAAAGAAATGCATAAGCTTTTGCCATTCTCACCGGATTCAGTCGTCACTCATGGTGATTTCTCACTTGATAACCTTATTTTTGACGAGGGGAAATTAATAGGTTGTATTGATGTTGGACGAGTCGGAATCGCAGACCGATACCAGGATCTTGCCATCCTATGGAACTGCCTCGGTGAGTTTTCTCCTTCATTACAGAAACGGCTTTTTCAAAAATATGGTATTGATAATCCTGATATGAATAAATTGCAGTTTCATTTGATGCTCGATGAGTTTTTCTAACACTCAGCTATCCAACTGATGCTGATAAATACCGCCTACAGGCACTTATTGCGCTGCAGGAGTTAGCAATTGAGCCGCGATCGCCGCTAGTTTTTGGGCCGCACCGGCCTCACCGCGAACCGCTCTGAGTTCCGCTTGCATGGCCGACAGTTTCTCTGGGTTCTGGAGATAGTCGAGAGCGATCGCGGCAATATCCTCGGGATAGTATTCGCCGATCAACTCGGGCACGATCGCCCGCTTCGCCCAAATATTCGGCCAAGCCAGCAGACCCACTTTGCGCAGCGCCAAGGTATTAATCAGGCTAGCGATCGCATTGCCCAACAGTGGCACCTTGACCAGCAAGCCCGGCAAGCCGTCCCAGGCTTTCATCGCATCCCGTTTTTGGGTTGGCAGCAGCACCAACATCGGCACCGCCAGCGCGCCTAATTCTGCTGTATTCGCGCCGATCGTGGTCAGGCAGATCTGGCACTGGGCCAACTCGACATAGGCTGGAAATTCCGTGATCAACCTTACGGTTAGCCCTTTTTCGGTCGTCAGCACCCAACCTGTGGGTTGCTCTTCTAAACGGGCGGAACTACCCCGAAACATTGCAATGACGGGATTTTGATCCGCATCCGCATAGCGAGCGATCTGCTGCGGTTGCACGGTCGGTGCGAGTGGCAGAATAAACTCCATCTCTGGTTGCTGAGCCGCGATCGC
